# Pandemics Throughout History

**DOI:** 10.3389/fmicb.2020.631736

**Published:** 2021-01-15

**Authors:** Jocelyne Piret, Guy Boivin

**Affiliations:** CHU de Québec – Laval University, Quebec City, QC, Canada

**Keywords:** infectious diseases, zoonotic pathogens, pandemic, public health measures, pharmaceutical interventions, water-borne pathogens

## Abstract

The emergence and spread of infectious diseases with pandemic potential occurred regularly throughout history. Major pandemics and epidemics such as plague, cholera, flu, severe acute respiratory syndrome coronavirus (SARS-CoV) and Middle East respiratory syndrome coronavirus (MERS-CoV) have already afflicted humanity. The world is now facing the new coronavirus disease 2019 (COVID-19) pandemic. Many infectious diseases leading to pandemics are caused by zoonotic pathogens that were transmitted to humans due to increased contacts with animals through breeding, hunting and global trade activities. The understanding of the mechanisms of transmission of pathogens to humans allowed the establishment of methods to prevent and control infections. During centuries, implementation of public health measures such as isolation, quarantine and border control helped to contain the spread of infectious diseases and maintain the structure of the society. In the absence of pharmaceutical interventions, these containment methods have still been used nowadays to control COVID-19 pandemic. Global surveillance programs of water-borne pathogens, vector-borne diseases and zoonotic spillovers at the animal-human interface are of prime importance to rapidly detect the emergence of infectious threats. Novel technologies for rapid diagnostic testing, contact tracing, drug repurposing, biomarkers of disease severity as well as new platforms for the development and production of vaccines are needed for an effective response in case of pandemics.

## Introduction

The shift from hunter-gatherers to agrarian societies has favored the spread of infectious diseases in the human population (Dobson and Carper, 1996). Expanded trades between communities have increased interactions between humans and animals and facilitated the transmission of zoonotic pathogens. Thereafter, expanded cities, extended trade territories, increased travels as well as effects on ecosystems due to increased human population raised the emergence and spread of infectious diseases leading to higher risks for outbreaks, epidemics and even pandemics (Lindahl and Grace, 2015).

The terms endemic, outbreak, epidemic and pandemic relate to the occurrence of a health condition compared to its predicted rate as well as to its spread in geographic areas (Grennan, 2019). An endemic condition occurs at a predictable rate among a population. An outbreak corresponds to an unpredicted increase in the number of people presenting a health condition or in the occurrence of cases in a new area. An epidemic is an outbreak that spreads to larger geographic areas. A pandemic is an epidemic that spreads globally.

An emerging infection newly appears in a population or is spread in a new geographic area (Morens et al., 2004). The zoonotic transmission of pathogens from animals to humans is a pivotal mechanism by which emerging infections have afflicted humans throughout history (Wolfe et al., 2007). The probability of cross-species transmission of pathogens was dramatically enhanced with increased interactions with animals through hunting, animal farming, trade of animal-based foods, wet markets or exotic pet trade (Bengis et al., 2004). The process of cross-species transmission of pathogens involves 5 different stages (Wolfe et al., 2007). (1) the pathogen exclusively infects animals under natural conditions; (2) the pathogen evolves so that it can be transmitted to humans but without sustained human-to-human transmission; (3) the pathogen undergoes only a few cycles of secondary transmission between humans; (4) the disease exists in animals but long sequences of secondary human-to-human transmission occur without the involvement of animal hosts; and (5) the disease occurs exclusively in humans. The animal species that harbor the pathogen, the nature of human interaction with that animal and the frequency of these interactions likely modulate the risk of zoonotic transmission. Furthermore, land use and climate changes are suggested to play important roles in the transmission of pathogens from wildlife to humans (El-Sayed and Kamel, 2020; White and Razgour, 2020). There is thus a need to implement surveillance programs to rapidly detect the emergence of pathogens with a potential for zoonotic transmission at the animal-human interface.

Climate changes also influence the transmission of pathogens (e.g., Dengue, Chikungunya, Zika, Japanese encephalitis, West Nile viruses, *Borrelia burgdorferi*) by expanding the habitats of various common zoonotic disease-carrying vectors (e.g., *Aedes albopictus* mosquitos, ticks) (Caminade et al., 2019). The emergence of vector-borne pathogens in non-endemic regions often results in explosive epidemics. Land use due to increasing human population also affects the distribution of disease-carrying vectors (Kilpatrick and Randolph, 2012). Control of vector-borne zoonotic pathogens usually requires vector control to reverse the drivers of transmission.

Furthermore, the spread of several infectious diseases (e.g., tuberculosis, malaria, cholera) to extended geographic areas are now raising health concerns for a significant proportion of the population (Morens et al., 2004). These diseases show a wider spread as a result of the acquisition of drug resistance, tolerance of mosquito vector to insecticides, poor sanitation, land use and climate changes as well as increased in human mobility and travels (Cutler et al., 2010). Furthermore, outbreaks of cholera in regions where natural disasters occurred such as earthquakes and floods were also reported. Surveillance programs should be also implemented to control the spread of these pathogens from endemic to non-endemic regions.

Finally, infectious agents (e.g., *Bacillus anthracis*, *Yersinia pestis*, variola virus) could be used as bioweapons and could thus constitute threats for humanity (Oliveira et al., 2020). These weapons are based on natural microorganisms or microorganisms that are engineered to be more virulent, highly transmissible or resistant to therapy (Narayanan et al., 2018). The release of these biological weapons is intended to induce diseases in humans or even death. Therefore, governments should establish biowarfare, bioterrorism and biocrime preparedness plans to protect the population.

In this paper, we review major pandemics that have afflicted humankind throughout history such as plague, cholera, influenza and coronavirus diseases, the way they were controlled in the past and how these diseases are managed today. Infectious diseases still represent threats for human health as pathogens can spread rapidly through global trade and travels. Global surveillance programs are thus needed to detect and identify pathogens spillover from animals to humans as well as to control water-borne pathogens and vector-borne diseases. Furthermore, effective non-pharmaceutical and pharmaceutical measures for the prevention and control of these infections are required to limit their dissemination in the human population.

## Plague

Plague is caused by the flea-borne bacteria *Yersinia pestis* that is responsible of at least three human plague pandemics, the plague of Justinian, the Black Death and the third plague (Table 1) (Zietz and Dunkelberg, 2004). *Y. pestis* is a gram-negative, rod-shaped, coccobacillus bacteria. *Y. pestis* is a facultative anaerobic bacteria that is transmitted by fleas associated mainly to rodents and other mammalian hosts (Perry and Fetherston, 1997). Fleas acquire the bacteria by sucking blood from an infected rodent. Bacteria quickly multiply and block the alimentary canal in the gut of the fleas (Bacot and Martin, 1914). The fleas transmit the bacteria to new rodent hosts by regurgitating the clotted blood. Plague manifests in three forms, i.e., bubonic, septicemic and pneumonic, depending on the route of infection (Yang, 2018). The bubonic form is the most common and results from the bite of an infected flea. Clinical manifestations include flu-like symptoms such as fever, chills, headache, body pains, weakness, vomiting and nausea followed by painful swollen lymph nodes. The bubonic form is likely to be fatal (50–90% of cases). Septicemic plague is rare (10–25% of cases) and consists of a progressive bloodstream infection in the absence of lymphadenopathy. The mortality rate is higher in patients with septicemic plague than in those with the bubonic form. Pneumonic plague occurs when the bacteria infects the lungs, either primarily by infectious respiratory droplets or secondarily as a complication of bubonic plague. This form is characterized by a fulminating onset and is rapidly fatal when left untreated.

**TABLE 1:** Timeline of the pandemics described in this paper.

Years	Pandemics	Pathogens	Vectors
541–543	Plague of Justinian	*Yersinia pestis*	Fleas associated to wild rodents
1347–1351	Black Death	*Yersinia pestis*	Fleas associated to wild rodents
1817–1824	First cholera pandemic	*Vibrio cholerae*	Contaminated water
1827–1835	Second cholera pandemic	*Vibrio cholerae*	Contaminated water
1839–1856	Third cholera pandemic	*Vibrio cholerae*	Contaminated water
1863–1875	Fourth cholera pandemic	*Vibrio cholerae*	Contaminated water
1881–1886	Fifth cholera pandemic	*Vibrio cholerae*	Contaminated water
1885–ongoing	Third plague	*Yersinia pestis*	Fleas associated to wild rodents
1889–1893	Russian flu	Influenza A/H3N8?	Avian?
1899–1923	Sixth cholera pandemic	*Vibrio cholerae*	Contaminated water
1918–1919	Spanish flu	Influenza A/H1N1	Avian
1957–1959	Asian flu	Influenza A/H2N2	Avian
1961-ongoing	Seventh cholera pandemic	*Vibrio cholerae*	Contaminated water
1968–1970	Hong Kong flu	Influenza A/H3N2	Avian
2002–2003	Severe acute respiratory syndrome (SARS)	SARS-CoV	Bats, palm civets
2009–2010	Swine flu	Influenza A/H1N1	Pigs
2015-ongoing	Middle East respiratory syndrome (MERS)	MERS-CoV	Bats, dromedary camels
2019-ongoing	COVID-19	SARS-CoV-2	Bats, pangolins?

*CoV, coronavirus; COVID-19, coronavirus disease 2019.*

### The Three Pandemics of Plague

The plague of Justinian occurred in Egypt and spread throughout the Eastern Roman Empire and its neighbors (Table 1) (Cunha and Cunha, 2008). Between 541 and 543, the plague killed an estimated 100 million people in the Roman Empire and especially in its capital, Constantinople. The highly developed structure of the Roman Empire facilitated the spread of the Justinian plague along its trade and military routes. In contrast, the plague did not affect the less organized barbarian societies outside of Rome’s borders. The high mortality caused by the disease might have contributed to the weakening and eventually to the decline of the Byzantine Empire. After this initial pandemic, intermittent plague outbreaks occurred every 8 to 12 years for two centuries and then disappeared for unknown reasons.

The identification of the causative pathogen involved in the death of victims of past pandemics usually relies on ancient DNA techniques aimed to directly extract DNA from skeletal remains. A great advance in paleomicrobiology techniques was the isolation of microbial DNA in dental pulp specimens (Drancourt et al., 1998). Indeed, bacteria are trapped in the dental pulp early in the course of a bacteremia and can be isolated from preserved teeth of victims. Therefore, analysis of the dental pulp is more efficient than bones to accurately identify microbial DNA from rapidly fatal infections that occurred in the past. Corpses of victims of the Justinian plague could be recovered in burial sites. Ancient DNA techniques from dental pulp samples identified *Y. pestis* as the etiological pathogen responsible for this pandemic (Harbeck et al., 2013).

The second plague pandemic, the Black Death, originated in East Asia and swept across Central Asia into Europe through the land and sea trade routes of the medieval Silk Road (Zietz and Dunkelberg, 2004). The second plague pandemic lasted in Europe until the early of the 19th century and killed 200 million people. The lineages of *Y. pestis* that caused the plague of Justinian and the Black Death were independent emergences into human population (Wagner et al., 2014). The Black Death (1347–1351) killed as many as 30% of the European population and was followed by successive waves such as the plague of Milan (1630), the great plague of London (1665–1666) and the plague of Marseille (1720–1722). It is suggested that the bacteria may have persisted in rodent reservoirs in Europe and periodically re-emerged in the human population (Seifert et al., 2016). Another hypothesis could be that climate-driven outbreaks of *Y. pestis* in Asian rodent reservoirs were responsible for new waves of plague arriving into Europe through its maritime trade network with Asia (Schmid et al., 2015). The bacteria suddenly disappeared from Europe and this could be possibly related to the extinction of local rodent reservoirs (Spyrou et al., 2016). At that time, there was no effective treatment against plague. Initial institutional responses to disease control began during the Black Death (Tognotti, 2013). A sanitary cordon was imposed by armed guards along transit routes and at access points to cities. A separation between healthy and infected persons was accomplished in camps and then in permanent plague hospitals (called lazarettos). Port cities were closed to ships arriving from plague-infected areas. Ships with suspicion of plague were put in quarantine, passengers and crew were isolated in lazarettos and vessels were thoroughly fumigated and retained for 40 days. The Black Death decimated Medieval Europe and had major impacts on its socio-economic development, culture, art, religion and politics (Bramanti et al., 2016).

Based on genomic analysis of ancient and recent genomes, it was suggested that a wave of plague may have traveled from Europe to Asia after the Black Death, eventually setting in China and giving rise to the third plague pandemic (Spyrou et al., 2016). The latter plague pandemic originated in the middle of the 19th century in the Yunnan region (China), reached Canton and spread to Hong Kong (Zietz and Dunkelberg, 2004). In 1894, Alexandre Yersin discovered the bacteria, *Y. pestis*, in specimens of plague patients and dead rats in Hong Kong (Yersin, 1894). The pandemic then reached Japan, Singapore, Taiwan and India via ships. Over the following years, plague became endemic in many countries around the world (Stenseth et al., 2008).

### The Plague Nowadays

Since the 1990’s, plague is classified as a re-emerging infectious disease by the World Health Organization (WHO) (World Health Organization [WHO], 2017a). Between 2010 and 2015, the number of plague cases was estimated at 3,248 with 584 fatalities worldwide, most of them occurring in Democratic Republic of the Congo, Madagascar and Peru (Glatter and Finkelman, 2020). In September 2017, a large outbreak of plague occurred in Madagascar with 2,417 cases among whom 77% were clinically suspected to have the pneumonic form (Mead, 2018). The basic reproduction number (R_o_) was estimated at 1.73 (Tsuzuki et al., 2017). The case fatality rate was as high as 8.6% (Mead, 2018). Plague is seasonal in most endemic countries with a well-defined geographic distribution which corresponds to those of the vectors and rodent reservoirs (Prentice and Rahalison, 2007). Nowadays, plague should be considered as a neglected human threat due to its rapid spread, its high fatality rate without early treatment and its capacity to disrupt social and healthcare systems (Valles et al., 2020). The genetic plasticity of *Y. pestis* also suggests a potential risk for the emergence of antibiotic resistance (Radnedge et al., 2002). Surveillance and control programs of fleas and animals involved in the life cycle of *Y. pestis* are required in endemic regions. Individuals should protect themselves against flea bites in regions where plague is present. It is also recommended to avoid contact with infected body fluids and tissues as well as animal carcasses. Public health interventions that can be put in place to prevent or limit plague outbreaks include the killing of fleas with insecticides and, if required, the control of infected rodents; the early isolation of patients, the rapid diagnostic and treatment of infected individuals with antibiotics; the detection and isolation of contacts and the administration of chemoprophylaxis to exposed individuals (Prentice and Rahalison, 2007). Standard treatments against plague include streptomycin and doxycycline. Alternative drugs consist of gentamicin and fluoroquinolones (Butler, 2014; Yang, 2018). The WHO does not recommend vaccination except for health care workers and laboratory personnel who are highly exposed to the pathogen. Fifty years ago, a plague vaccine based on whole *Y. pestis* killed with formalin was approved in the United States. This vaccine was effective against bubonic plague but not against the pneumonic form of the disease. Furthermore, this vaccine was associated with a high reactogenicity. As highlighted in the Madagascar outbreak, *Y. pestis* can be responsible for pneumonic plague in a high number of cases. Furthermore, *Y. pestis* can be aerosolized for use as a bioweapon. *Y. pestis* is considered as a category A pathogen as it can be easily disseminated or transmitted from person to person, cause high mortality rates and have the potential for major public health impact (Ansari et al., 2020). Therefore, the development of a plague vaccine against the most deadly form of the disease is needed. In 2017, the US Food and Drug Administration (FDA) granted Orphan Drug designation to the recombinant F1 and V subunits vaccine (rF1V) that is intended as a prophylactic vaccine for individuals at high-risk of exposure to aerosolized *Y. pestis*. Several prophylactic and therapeutic plague vaccines are also being developed under the Plague Vaccine Target Product Profile (TPP) established by the WHO in 2018 (World Health Organization [WHO], 2018a). Vaccines under development include subunit, bacterial vector-based or viral vector-based vaccines expressing one or several antigens [F1 capsular protein, low calcium response protein V (LcrV), YscF and/or pesticin coagulase] of *Y. pestis* (Sun and Singh, 2019).

## Cholera

Cholera is an acute often fatal disease of the gastrointestinal tract caused by *Vibrio cholerae* (Faruque et al., 1998). *V. cholerae* is a gram-negative and coma-shaped bacteria. *V. cholerae* is a facultative anaerobe and has a flagellum at one cell pole as well as pili. The bacteria colonizes the small intestine and produces the cholera toxin which is responsible for a rapid and massive loss of body fluids leading to dehydration, hypovolemic shock and death. *V. cholerae* strains are classified based on their major lipopolysaccharide O antigens into approximately 206 serogroups of which serogroups O1 (consisting of two biotypes known as classical and El Tor) and O139 cause epidemic cholera (Chatterjee and Chaudhuri, 2006). *V. cholerae* is a water-borne pathogen. Humans are infected through contaminated water used for drinking or preparing foods. The infection is often mild or asymptomatic and bacteria are eliminated with feces in 1 or 2 weeks. *V. cholerae* persists indefinitely in aquatic reservoirs and may acquire mobile elements via horizontal gene transfer leading to the emergence of new toxigenic clones (Cho et al., 2010). *V. cholerae* are able to form a three-dimensional biofilm where the organisms can survive during inter-epidemic periods (Alam et al., 2007). Optimal growth conditions of bacteria in aquatic reservoirs include 15% salinity, 30°C water temperature and pH 8.5 that can be promoted by climate changes (Montilla et al., 1996).

### The Seven Cholera Pandemics

Cholera was endemic in Asia until 1817, when a first pandemic spread from India to several other regions of the world (Table 1) (Faruque et al., 1998). This pandemic emerged during a period of increasing globalization resulting from technological progress in transportation. Indeed, the advent of steamships and railways allowed a dramatic decrease in travel time and a rise in trade. At that time, health prevention strategies were essentially the same than those implemented during the Black Death (Tognotti, 2013). Infected persons were isolated in lazarettos. Port entry was forbidden for ships arriving from regions where cholera was present. Travelers who had contacts with infected persons or who came from a place where cholera was circulating were quarantined.

Thereafter, five additional major pandemics of cholera that originated from India and spread to other continents occurred during the 19th and 20th centuries (Table 1) (Faruque et al., 1998). The second and sixth pandemics, and presumably the other ones, were caused by the O1 classical biotype of *V. cholerae* (Devault et al., 2014; Siddique and Cash, 2014). The second pandemic of cholera reached the British islands. During the cholera outbreak in Soho (London), in 1854, the physician John Snow used for the first time epidemiological methods to trace the source of the outbreak. He described the time course of the outbreak and its geographical spread in the city. He identified the public pumps used for water supply in these areas and understood that water was the source of the contamination. He then proposed effective measures to prevent the transmission through the removal of pump handle in the city areas where the outbreak occurred (Smith, 2002). The bacillus of cholera was isolated during the fifth pandemic that extensively affected South America by Robert Koch (1884) who also understood the importance of clean water in preventing its transmission. The toxin responsible for the disease was only discovered in 1959 (De, 1959). The seventh cholera pandemic is the most extensive in terms of geographic spread and duration (Mutreja et al., 2011; Hu et al., 2016). It began in Indonesia in 1961 and became endemic in many regions of the world. It periodically caused major epidemics such as those in Zimbabwe (2008), Haiti (2010), Sierra Leone (2012), Mexico (2013), South Soudan and Ghana (2014) and Yemen (2016). Cholera epidemics usually ended because of a lack of favorable environmental conditions for the survival of vibrios. The seventh cholera pandemic was caused by El Tor biotype strain of *V. cholerae* that acquired virulence genes in the environment (Safa et al., 2010). In late 1992, the O139 serogroup caused a large cholera outbreak in Bangladesh and neighboring countries and raised the fear of a 8th cholera pandemic (Albert et al., 1993).

### Cholera Nowadays

Cholera cannot be eradicated as it is a natural inhabitant of aquatic ecosystems. *V. cholerae* serogroups O1 and O139 are responsible for cholera outbreaks in developing countries. In 2019, 923,037 cases and 1,911 deaths were reported to WHO in 31 countries (World Health Organization [WHO], 2020d). However, it is estimated that the global burden of cholera is higher due to underreporting. Between 2008 and 2012, the number of cases was shown to range between 1.3 and 4 millions per year with 95,000 deaths (Ali et al., 2015). Environmental and climate changes may increase the geographical distribution of cholera (Chowdhury et al., 2017). Furthermore, the emergence of environmental non-O1/O139 *V. cholerae* is increasing as genetic exchange mechanisms such as horizontal gene transfer and genetic recombination are favored by changes in ecosystem and climate (Vezzulli et al., 2020). These non-O1/O139 vibrios can cause illness that can be mild (e.g., gastroenteritis, otitis) to life-threatening (necrotizing fasciitis) and could thus constitute a potential threat for humans. The persistence of cholera is related to poor living conditions including shortage of safe drinking water, insufficient sanitation, crowded housing and the lack of efficient sewage systems. Re-emergence of the disease can also occur following natural disasters such as earthquakes that disrupt access to safe water supply. Cholera outbreaks could be predicted based on real-time monitoring of oceanic regions, climate fluctuations and epidemiological surveillance program (Chowdhury et al., 2017). The disease could be prevented by implementation of public health measures to ensure adequate sanitation and safe water supply (Somboonwit et al., 2017). The access to safe drinking water and sanitation are among the primary priorities of the Millenium Development Goals and the sustainable Development Goals (Igere and Ekundayo, 2020). Furthermore, the Water, Sanitation and Hygiene (WASH) program launched by the WHO is central to the prevention of cholera transmission (Wolfe et al., 2018). Currently, three oral cholera vaccines were pre-qualified by the WHO (Dukoral^®^, Sanchol^TM^ and Euvochol-Plus^®^). These vaccines should be used in areas with endemic cholera, cholera outbreaks and humanitarian crisis with high-risk of cholera in combination with other cholera prevention and control measures (World Health Organization [WHO], 2019). Cholera is first identified based on clinical symptoms of severe acute watery diarrhea. The disease is then confirmed by the detection of *V. cholerae* in stool samples by culture, molecular testing or rapid diagnostic tests. The majority of infected individuals can be treated by the administration of prompt oral rehydration solution. Severely dehydrated patients who are at risk of shock require rapid administration of intravenous fluids as well as antibiotics. First-line drug consists of doxycycline whereas alternative treatments include tetracycline, ciprofloxacin and azithromycin (Hsueh and Waters, 2019).

## Influenza

Influenza viruses belong to the *Orthomyxoviridae* family. Influenza viruses are enveloped, negative-sense, single-stranded RNA viruses (Wright and Webster, 2001). Their genome consists of 7 or 8 RNA segments encoding at least 10 structural and non-structural proteins. Structural proteins include a hemagglutinin (HA), a neuraminidase (NA), two matrix proteins and a nucleoprotein. Influenza viruses can be distinguished in types A, B, C, and D. Influenza A and B are responsible for outbreaks in tropical regions and seasonal epidemics in temperate regions whereas influenza A viruses are the only ones with a pandemic potential (Lofgren et al., 2007). Indeed, influenza A virus is endemic in a number of species including humans, birds and pigs (Webster et al., 1992). Gene reassortments can thus occur between human and animal influenza A viruses and lead to a new virus subtype which can be pathogenic to humans (Webster et al., 1995).

In a typical seasonal epidemics, influenza virus causes 3 to 5 million cases of severe illness and approximately 500,000 deaths worldwide (Iuliano et al., 2018). Most typical seasonal influenza infections are asymptomatic or cause only mild or classical influenza illness characterized by 4 or 5 days of fever, cough, chills, headache, muscle pain, weakness and sometimes upper respiratory tract symptoms (Zambon, 2001). Severe complications can occur especially in infants, elderly and individuals with chronic conditions such as diabetes mellitus and cardiac/pulmonary diseases. Among the most severe complications is pneumonia which can be associated with secondary bacterial infection.

Annual influenza epidemics are sustained in the human population through mutations occurring especially in the HA and NA viral surface glycoproteins, the major targets for neutralizing antibodies. Seasonal influenza virus results from frequent antigenic drifts every 2–5 years in response to selection pressure to evade human immunity (Kim et al., 2018). Its genome contains segmented genes which may undergo reassortments in cells co-infected with two or more influenza viruses. Each influenza A virus has a gene encoding for 1 of 16 possible HAs and another gene encoding for 1 of 9 possible NAs that are involved in viral attachment and release, respectively (Dugan et al., 2008). Of the 144 total combinatorial possibilities, only 3 HAs and 3 NAs in only 4 combinations (A/H1N1, A/H2N2, A/H3N2 and possibly A/H3N8) were found to be truly adapted to humans. Rarely, antigenic shift which results from reassortment between human and animal viruses leads to the emergence of a new virus subtype (Webster et al., 1995; Ma et al., 2009). This antigenically distinct virus may have the ability to infect humans and achieve sustained human-to-human transmission and may cause a pandemic if the immunity in the human population is partial or lacking (Webster et al., 1992).

### Influenza Pandemics

The time in which influenza virus began to infect humans or cause a pandemic cannot be determined with accuracy but many historians agree that the first influenza pandemic could have likely occurred in 1510 (Morens et al., 2010). The Russian flu that occurred between 1889 and 1893 was the first well-described pandemic (Taubenberger et al., 2007). This pandemic was possibly caused by an A/H3N8 virus based on serologic and epidemiologic data (Worobey et al., 2014). The virus spread rapidly as it took only 4 months to circumvent the planet (Valleron et al., 2010). The pandemic virus reappeared every year for 3 years and caused an estimated 1 million deaths worldwide (Table 1). The median R_o_ was estimated at 2.1 (interquartile range 1.9–2.4) (Valleron et al., 2010). The case fatality rates ranged from 0.10 to 0.28% so the mortality burden of this pandemic was considered as low (Valleron et al., 2010). The median clinical attack rate was 60% (interquartile range 45–70%) (Valleron et al., 2010). Attack rates were highest in individuals aged 1–60 years and lower in infants and seniors (Valtat et al., 2011). In contrast, mortality rate showed a J-shape curve with highest rates in infants and people over 20 years of age (Valtat et al., 2011).

Twenty five years later, the Spanish flu was caused by an A/H1N1 virus (Table 1) that apparently arose by genetic adaptation of an existing avian influenza virus to a new human host (Reid et al., 2004). Before its identification, the virus spread silently around the world and its region of origin could not be determined. Analysis of formalin-fixed and paraffin-embedded samples as well as permafrost-frozen corpses from that time confirmed that the strain was an A/H1N1 influenza virus (Reid et al., 1999). Typical attack rates were 25–33% and the R_o_ was estimated at 2–3 (Mills et al., 2004). The 1918–1919 pandemic spread in at least 3 distinct waves within a 9 month interval. The first wave occurred during spring-summer 1918 and caused high morbidity and low mortality. Both the second and third waves in summer-fall 1918 and winter 1918–1919 caused high mortality. The 1918–1919 influenza pandemic resulted in approximately 500 million infections and 50 million deaths worldwide (Johnson and Mueller, 2002). Fatality of epidemic influenza usually follows a characteristic U-shape curve with high mortality rates in the very young (< 5 years) and the elderly (> 65 years). However, the 1918–1919 pandemic showed a W-shaped mortality curve with high case fatality rates in the very young and the elderly as well as in healthy young adults aged 20–40 years (Morens and Taubenberger, 2018). This uncommon age distribution suggests that the severity of the 1918–1919 influenza pandemic was not primarily due to a hyper-virulent strain but was more likely related to host factors that prevent individuals to control the infection. It is suggested that an A/H3N8 virus was circulating between 1890 and 1900 and that individuals born at this time may have lacked immunity against the antigenically distinct 1918 A/H1N1 virus (Worobey et al., 2014). The most common clinical manifestation was an acute aggressive bronchopneumonia presenting with epithelial necrosis, microvasculitis/vascular necrosis, hemorrhages, edema and severe tissue damage to the lungs (Morens and Fauci, 2007) often followed by secondary bacteria invasion with *Streptococcus pneumonia*, *Streptococcus pyogenes*, *Staphylococcus aureus* and *Haemophilus influenzae* (Morens et al., 2008). It is suggested that the 1918 influenza virus had an enhanced capacity to spread to and damage bronchial and bronchiolar epithelial cells that could allow bacteria to breach the mucociliary barrier leading to fatal bacterial pneumonia (Morens and Fauci, 2007). The second common clinical manifestation was an acute respiratory distress syndrome (ARDS) associated with severe facial cyanosis that was observed in 10–15% of cases (Shanks, 2015). The H1 hemagglutinin of the 1918–1919 pandemic virus was identified as a key virulence factor for mammalian and was associated with increased respiratory epithelial pathogenicity and elicitation of a strong pro-inflammatory response (Qi et al., 2014). Most deaths occurred from several days to weeks (median 7–10 days) after the onset of symptoms (Shanks and Brundage, 2012). In large cities of Western world, health authorities implemented a series of containment strategies to prevent the spread of the disease including the closure of schools, churches and theaters and the suspension of public gatherings. Physicians encouraged the practice of individual measures such as respiratory hygiene and social distancing. However, these measures were implemented too late and in an uncoordinated manner due to World War I. Travel restrictions and border controls were impossible to put in place. The movement of military troops and the poor living conditions of soldiers in the trench warfare in Europe facilitated the spread of the disease.

**FIGURE 1:**
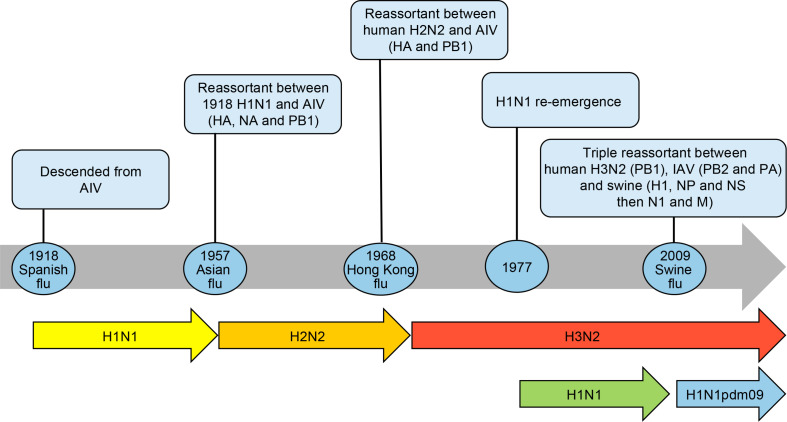
Timeline of influenza pandemics caused by the 1918 H1N1 virus and its descendants produced by reassortment of circulating strains with avian influenza viruses (AIV) and swine H1N1 viruses. The reassortment of genes is shown in parenthesis. The re-emergence of H1N1 virus in 1977 is also shown as it co-circulated with the H3N2 virus before being replaced by the H1N1pdm09. HA, hemagglutinin; NA, neuraminidase; NP, nucleoprotein; M, matrix proteins, PB1 polymerase; PB2 polymerase; PA polymerase; NS, non-structural proteins.

Over the past century, descendants of the 1918 pandemic virus were the cause of almost all seasonal influenza A epidemics worldwide. All influenza A viruses responsible for the 1957, 1968 and 2009 pandemics (Table 1) also derived from the founding 1918 virus by gene reassortments between human, avian and swine influenza viruses (Morens et al., 2009) as shown in Figure 1.

The new subtype A/H2N2 that caused the 1957–1959 pandemic (Asian flu) derived from the 1918 virus by acquisition of 3 new avian gene segments (HA, NA and PB1 polymerase) by reassortment (Kawaoka et al., 1989) whereas the circulating A/H1N1 was totally replaced. Sustained transmission of the 1957–1959 pandemic virus started on December 1957 with recurrent waves occurring over several years (Housworth and Langmuir, 1974). The morbidity was highest in children and the mortality was highest at the extremes of age. The case fatality rate was approximately 0.13% (Mc, 1958). The global mortality of the 1957–1959 influenza pandemic was estimated at 1–2 millions based on excess death due to respiratory diseases (Viboud et al., 2016). The R_o_ was estimated at 1.65 (interquartile range 1.53–1.70) (Biggerstaff et al., 2014). The highest attack rates were in school-age children through young adults up to 35 or 40 years of age (Serfling et al., 1967). Older adults, including those over the age of 60, had significantly lower attack rates. This unusual distribution was attributed to the absence of protective antibody among children and middle-aged adults. Histopathological studies from autopsies were characterized by a rapid development of bronchial epithelial necrosis, preservation of the basal layer, limited inflammatory response and evidence of prompt repair (Walsh et al., 1961). Secondary bacterial pneumonia was a relatively minor cause of fatalities possibly as a result of the widespread use of antibiotics (Robertson et al., 1958; Oseasohn et al., 1959). The proportion of strains resistant to antibiotics was relatively high in fatal cases compared to those isolated from cases who recovered. Furthermore, mechanical ventilators were available in the intensive care units (ICU) to support cases presenting hypoxemia. At that time, the pathogenic agent had been identified (Smith and Andrewes, 1933) and knowledge on the pathogenesis of the disease had advanced. In 1952, the WHO had implemented a global influenza surveillance network that provided information on the emergence and spread of the novel influenza virus. Containment measures (such as closure of schools and nursery, bans on public gatherings) varied from country to country but delayed the onset of the disease for a few weeks only. Influenza vaccination (vaccine efficacy of 53–60%) failed to have a significant impact on the pandemic due to inadequate coverage (Henderson et al., 2009).

The 1968–1970 pandemic virus emerged when 2 gene segments encoding for HA and PB1 polymerase moved from an avian virus in the A/H2N2 virus through genetic reassortment whereas the NA remained unchanged (Kawaoka et al., 1989). The new A/H3N2 reassortant replaced the A/H2N2 virus that circulated in the human population since 1957. The global mortality rate of the 1968–1970 pandemic (Hong Kong flu) was estimated to be 0.5–2 millions (Saunders-Hastings and Krewski, 2016). The R_o_ was estimated at 1.80 (interquartile range 1.56–1.85) (Biggerstaff et al., 2014). The mean age at death was 62–65 years. The first pandemic season was more severe than the second one in North America whereas the opposite was seen in Europe and Asia (Viboud et al., 2005). The 1968–1970 influenza pandemic was mild in all countries and comparable to severe seasonal epidemics. The mildness of this pandemic is expected considering pre-existing immunity to the NA antigen in all age groups and to the HA in the elderly. No specific containment measures were implemented during this pandemic. In 1977, a descendant of the 1918 A/H1N1 re-emerged suspiciously in Russia and co-circulated with the reassortant A/H3N2 virus thereafter (Gregg et al., 1978).

The 2009 A/H1N1 pandemic was a triple reassortant made of influenza genes originating from human A/H3N2 (PB1 polymerase gene segment), avian (PB2 polymerase and PA polymerase gene segments) and North American (H1, nucleoprotein and Non-structural proteins gene segments) and Eurasian (N1 and matrix proteins gene segments) swine that was transmitted from pigs to humans (Easterbrook et al., 2011). The 2009 H1 protein had minimal antigenic drift compared to its 1918 counterpart. Due to its pathogenicity in humans, it is suggested that the maintenance of H1 immunity in the population may be important to prevent future pandemics (Morens and Taubenberger, 2018). The 2009 influenza virus emerged in Mexico and almost simultaneous outbreaks began in Mexico and in Southern United States (Neumann and Kawaoka, 2011). The virus then spread globally over the next 6 weeks. The A/H1N1pdm09 virus was associated with a lower attack rate in older individuals possibly because of previous exposition to older A/H1N1 viruses. Clinical symptoms associated to the A/H1N1pdm09 range from mild respiratory irritations to severe pneumonia associated to ARDS when infection progressed (Chowell et al., 2009). Asymptomatic infections accounted for approximately 10% of cases (Papenburg et al., 2010). Severe disease developed in a small proportion of healthy adults, many of whom had no underlying conditions (Viboud et al., 2010). The R_o_ was estimated at 1.46 (interquartile 1.30–1.70) (Biggerstaff et al., 2014). The WHO reported 18,631 laboratory-confirmed deaths. However, the mortality burden was estimated to be between 148,000 and 249,000 based on excess death due to respiratory diseases in several countries (Simonsen et al., 2013). The case fatality rate based on confirmed cases was 0.5% (Nishiura, 2010). Later studies estimated the symptomatic case fatality rate at 0.05% of all medically attended symptomatic cases. Mortality rates in younger populations affecting children, young adults and pregnant women were higher than in a typical influenza season. The average age of people who died with laboratory-confirmed influenza was 37 years (Vaillant et al., 2009). Non-pharmaceutical interventions that were implemented included hand washing, use of face masks and cough etiquette (Cantey et al., 2013). The 2009 pandemic was the first one to combine vaccines and antiviral use. Symptomatic individuals and their contacts were isolated and received antiviral treatment as prophylaxis. Although the vaccine was approved only during the second wave, the immunization program in Canada covered 33 to 50% of its population compared to 13 to 39% in United States (Gilmour and Hofmann, 2010). The new pandemic virus completely replaced the prior circulating seasonal A/H1N1 whereas the influenza A/H3N2 continued to circulate.

Overall, the impacts of an influenza pandemic depend on the transmissibility and virulence of the strain and on the susceptibility of the population, which may vary according to age and past exposure to influenza viruses. The impacts of influenza are not always higher during pandemics than during seasonal epidemic periods. However, a shift in the age distribution of mortality toward younger age groups distinguishes the impacts of a pandemic from those of seasonal epidemics (Simonsen et al., 1998).

### Avian Influenza and the Risks for a New Pandemic

The constant adaptation and exchange of genes between influenza viruses in different species, including at the animal-human interface, is still a critical challenge for the emergence of pandemic viruses nowadays. In this respect, a series of avian influenza A viruses have caused sporadic cases and outbreaks of severe diseases and deaths in humans (Li et al., 2019). These viruses are divided into two groups, low pathogenic avian influenza (LPAI) and highly pathogenic avian influenza (HPAI) viruses, based on their virulence in chicken. The first human outbreak due to a HPAI was caused by an A/H5N1 virus in 1997 in Hong Kong where 18 positive cases associated with 6 deaths were reported (Chan, 2002). This A/H5N1 HPAI virus continues to spread in poultry and in a large number of wild bird species on several continents. This virus caused severe and fatal spillover infections in humans and rarely resulted in human-to-human transmission (Ungchusak et al., 2005). This virus was eventually detected in 17 countries and led to 861 human cases with a case fatality rate of 53% as of October 23, 2020 (World Health Organization [WHO], 2020b). The A/H5N1 virus has thus a potential for high morbidity and mortality in humans but it seems unlikely that it could become adapted with efficient human-to-human transmission (Morens and Taubenberger, 2015). A LPAI A/H7N9 virus emerged in China in 2013 (Gao et al., 2013). This virus was then shown to evolve to highly pathogenic strains in late 2016 (Kile et al., 2017). Infection with A/H7N9 has been reported in 1,567 human cases with a fatality rate of 39% as of September 5, 2018 (World Health Organization [WHO], 2018b). To date, sporadic familial clusters of A/H7N9 have been reported but there is still no evidence of sustained human-to-human transmission of the virus (Wu et al., 2020). Sporadic cases of human infections with avian influenza viruses also occurred with A/H5N6, A/H6N1, A/H7N2, A/H7N3, A/H7N4, A/H7N7, A/H9N2, A/H10N7 and A/H10N8 strains (Widdowson et al., 2017). Surveillance programs for monitoring animal influenza viruses with zoonotic potential facilitate the rapid detection of human threats. However, obvious clinical manifestations of influenza infections may be lacking in avian species thereby complicating early detection and efficient control of potential outbreaks (Li et al., 2019). Furthermore, the conditions required for cross-species transmission from avian species to humans are not yet elucidated and surveillance programs would most likely require longitudinal surveillance in multiple hosts. Non-pharmaceutical measures aim to reduce the number of live-bird markets and to decrease contacts between humans and birds in breeding facilities have been implemented to prevent and control zoonotic influenza virus infections. Animal facilities must be periodically disinfected and employees exposed to birds must wear protective personal equipment and be isolated in case of suspected contamination. Pharmaceutical measures include the use of vaccines (including poultry vaccination) and antiviral agents such as the neuraminidase inhibitors (oseltamivir, zanamivir and peramivir) and polymerase inhibitors (baloxavir marboxil and favipiravir) (Beigel and Hayden, 2020). The WHO together with reference laboratories determine viral antigenicity of strains circulating in avian species that could be used in the development of candidate vaccines for pandemic preparedness. To date, candidate vaccines are available for H5, H7 and H9 influenza viruses (World Health Organization [WHO], 2020a). The development of a universal vaccine to prevent any subtype of influenza virus is a priority (Yamayoshi and Kawaoka, 2019). Finally, it is not excluded that the circulating human A/H3N2 virus could acquire an avian H2 gene by reassortment. As the majority of the population has no protective immunity against the H2 subtype that circulated between 1957 and 1968, the emergence of a H2N2 reassortant could be a potential risk for a future pandemic (Taubenberger and Morens, 2010).

## Coronaviruses

Coronaviruses belong to the *Coronaviridae* family and include four genera, i.e., alpha-, beta-, gamma-, and delta-coronaviruses (Masters and Perlman, 2013). Coronaviruses are enveloped, positive-sense, single-stranded, RNA viruses that infect a wide range of animals and humans. The genome encodes non-structural proteins and 4 structural proteins including the membrane, spike, envelope and nucleocapsid proteins. Human coronaviruses (HCoVs) cause seasonal respiratory diseases, and to a lesser extent, gastroenteritis. HCoV-229E (alpha-coronavirus) and HCoV-OC43 (beta-coronavirus) are the causative agents of common cold (Kahn and McIntosh, 2005). HCoV-NL63 (alpha-coronavirus) and HCoV-HKU1 (beta-coronavirus) cause more severe, although rarely fatal, infections of the upper and lower respiratory tracts (Kahn and McIntosh, 2005). Furthermore, beta-coronaviruses also include three highly pathogenic viruses such as severe acute respiratory syndrome coronavirus (SARS-CoV), Middle East respiratory syndrome coronavirus (MERS-CoV) and SARS-CoV-2 [the etiological agent of coronavirus disease 2019 (COVID-19)] that induce severe pneumonia in humans (Table 1) (Song et al., 2019).

### SARS-CoV Epidemic

SARS-CoV originated in Guangdong province (China) in 2003. Bats are likely the possible natural reservoir of SARS-CoV (Li et al., 2005) and palm civets could be intermediary hosts before dissemination to humans (Guan et al., 2003). The causative agent was identified within a few weeks (Drosten et al., 2003; Ksiazek et al., 2003). During the 2002–2003 outbreak, SARS-CoV infection was reported in 29 countries in North America, South America, Europe and Asia. Overall, 8,437 probable cases were reported with 813 SARS-related fatalities (World Health Organization [WHO], 2003). The case fatality rate was 9.7%. Transmission of SARS-CoV was mainly nosocomial with rates of 33–42% whereas rates of 22–39% occurred between family members (Chowell et al., 2015). Infection with SARS-CoV typically caused an influenza-like syndrome with rigors, fatigue and high fever. Less common symptoms include nausea, vomiting and diarrhea. In 20–30% of infected patients, the disease progressed to an atypical pneumonia, with shortness of breath and poor oxygen exchange in the alveoli with the patients requiring management in ICU or mechanical ventilation. Many of these patients also developed watery diarrhea with active virus shedding. Respiratory failure was the most common cause of death among patients infected with SARS-CoV. The major routes of transmission of SARS-CoV were droplets, aerosols and fomites (Seto et al., 2003). The R_o_ of SARS-CoV was approximately 3 (Petersen et al., 2020). SARS-CoV quickly became a global threat because of its rapid transmission and high mortality rate. Protective immunity against this virus as well as effective antiviral drugs and vaccines were lacking. The low infectivity and long incubation period (viral load peaks at 6–11 days after symptom onset) of SARS-CoV provided time for implementing a series of containment measures to prevent transmission (Weinstein, 2004). Case identification and isolation followed by contact tracing and surveillance proved effective in containing the global threat and eradicating the virus in almost 7 months. However, some SARS-CoV-like viruses found in bats have been shown to be able to infect human cells without prior adaptation, which indicates that SARS could re-emerge in the future (Ge et al., 2013).

### MERS-CoV Epidemic

Ten years after the first emergence of SARS-CoV, MERS-CoV was reported in Jeddah in Saudi Arabia. The potential animal reservoirs of MERS-CoV are bats and dromedary camels have been suggested as the intermediary hosts (Conzade et al., 2018). Advances in molecular diagnostic tools such as next-generation sequencing allowed that the etiological agent was identified within weeks of global spread (Zaki et al., 2012). Between 2012 and 2020, 2,519 laboratory-confirmed cases of MERS-CoV with at least 866 deaths were reported in 27 countries (World Health Organization [WHO], 2020c). All cases have been linked to persons in the Arabian Peninsula or who had returned from traveling in MERS-CoV endemic areas. Almost 50% of MERS-CoV cases were due to nosocomial transmission to inpatients, health care workers and visitors (Hui et al., 2018). Transmission between family members has only occurred in 13–21% of cases (Chowell et al., 2015). Individuals with MERS-CoV present with a wide range of clinical features from mild to severe fulminant pulmonary disease (Memish et al., 2020). Those with severe disease are often older than 65 years with comorbidities and can develop symptoms at a later time. Asymptomatic to mild infection rates of 25–50% have been reported. Infection with MERS-CoV causes acute, highly lethal pneumonia and renal dysfunction with various clinical symptoms including fever, chills, rigors, headache, a non-productive cough, sore throat, arthralgia and myalgia. Other symptoms include nausea, vomiting, diarrhea and abdominal pain. It is estimated that 50–89% of patients with progression to respiratory and/or renal failure require ICU admission. The high incidence of ARDS in these patients is reflected by a high case fatality rate of 34%. The R_o_ of MERS-CoV is low (approximately 1) (Petersen et al., 2020) which allows to control the transmission in the absence of mitigation strategies, although the virus caused several nosocomial outbreaks in hospital of Saudi Arabia, Jordan and South Korea.

MERS-CoV is still circulating nowadays. The ubiquity of infected dromedary camels close to humans (Kandeil et al., 2019) and the continuing zoonotic transmission may explain why MERS-CoV continues to cause intermittent sporadic cases, community clusters and nosocomial outbreaks (Sikkema et al., 2019). Furthermore, the outbreak in South Korea highlights the potential of MERS-CoV to spread around the world and thus to be a threat for global health (Petersen et al., 2015). There is currently no licensed vaccine or treatment available against MERS-CoV infection. The clinical management of patients with MERS-CoV consists mainly in providing supportive care for the relief of pain and fever, supporting vital organ functions and treating concomitant or secondary bacterial infections with antibiotics (Memish et al., 2020). Critically ill patients required to be managed in an ICU. The WHO is supporting molecular and serological surveillance studies at the dromedary-human interface in several Middle East countries (World Health Organization [WHO], 2018c). Indeed, recombination events were reported to occur leading to different lineages of MERS-CoV in dromedary camels (Sabir et al., 2016). New MERS-CoV variants were also shown to emerge over time (AlBalwi et al., 2020). Furthermore, this organization has issued recommendations for not consuming unpasteurized camel milk and undercooked animal products and to be cautious in cases of close contacts with dromedary camels. Appropriate hospital hygiene practices and implementation of contact and droplet precautions are crucial to limit future nosocomial outbreaks. The implementation of extensive contact tracing in order to rapidly diagnose suspected MERS-CoV cases and isolation of individuals to break the chain of infections in the community is also essential. The WHO calls for the development of three types of vaccines against MERS-CoV: a human vaccine that is intended for long-term protection of individuals at high-risk of exposure such as health care workers and those having contacts with potentially infected dromedary camels, a human vaccine for use during outbreaks and a dromedary camel vaccine to prevent zoonotic transmission (World Health Organization [WHO], 2017b). The spike protein of MERS-CoV is an important target for the development of vaccines (Zhang N. et al., 2020). These vaccines are based on DNA, viral vectors, viral proteins, virus-like particles, bacterium-like particles and nanoparticles. The progression of most of these vaccines from pre-clinical studies to clinical trials are prevented by lack of funds. Only a few ones were tested in phase I clinical studies until now.

### SARS-CoV-2 Pandemic

In early December 2019, atypical pneumonia were reported in a cluster of patients in Wuhan (China) and were shown to be caused by a new coronavirus, called SARS-CoV-2 (Zhu et al., 2020) whereas the disease is referred to as COVID-19. The animal reservoirs are likely bats (Lau et al., 2020). It was proposed that pangolins could be the animal hosts that transmit the virus to humans (Lam et al., 2020) but the intermediary host, if any, has not yet been definitely identified. SARS-CoV-2 infection may be asymptomatic (up to 40% of cases) or causes a wide spectrum of illness from mild symptoms to life-threatening disease (Wiersinga et al., 2020). Infected individuals most commonly present with fever, dry cough, shortness of breath, fatigue, myalgia, nausea/vomiting or diarrhea, headache, weakness, rhinorrhea, anosmia and ageusia. Common complications among hospitalized patients include pneumonia, ARDS, acute liver injury, cardiac injury, prothrombotic coagulopathy, acute kidney injury and neurologic manifestations. Critically ill patients could also develop a cytokine storm and a macrophage activation syndrome. Comorbidities such as hypertension, diabetes, cardiovascular disease, chronic pulmonary disease, chronic kidney disease, malignancy and chronic liver disease have been present in 60–90% of hospitalized patients (Richardson et al., 2020). Mild symptoms occurred in 80% of laboratory-confirmed cases. Approximately 14–19% of patients are hospitalized and 3–5% of cases require transfer to ICU, most commonly due to hypoxemic respiratory failure. Among them, 29–91% necessitate invasive mechanical ventilation. Overall, mortality of hospitalized patients with COVID-19 is approximately 15–20% whereas it is as high as 40% in patients requiring ICU admission. The global estimated case fatality rate ranges between 0.25 and 3.0% (Wilson N. et al., 2020). Mortality ranges from 0.02% in patients aged 20–49 years to 0.5% for patients aged 50–69 years and greater than 5.4% for patients aged more than 80 years. Children with COVID-19 have milder symptoms predominantly limited to the upper respiratory tract. However, a rare multisystem inflammatory syndrome has been described in some children with COVID-19 (Rowley et al., 2020). Impaired type I interferon response was shown to be involved in patients with life-threatening COVID-19 (Bastard et al., 2020; Zhang Q. et al., 2020). The R_o_ is estimated to be between 2 and 3 (Petersen et al., 2020). COVID-19 has spread globally within a few months leading to more than 74 million contaminations and more than 1.6 million deaths worldwide as of December 18, 2020 (World Health Organization [WHO], 2020e).

Multiple public health measures have been implemented by most countries such as individual actions (physical distancing, hand washing, use of face masks and cough etiquette), identification of clusters (case identification, contact tracing and isolation), regulatory actions (school closure, workplace closure, stay-at-home order, public transport closure and restriction, limits on size of gatherings and business capacity), closures of internal and international borders, travel restrictions and enforced quarantine. The goal of these public health measures being to delay and flatten the epidemic curve, preventing overwhelming capacity of the health care system and protecting individuals at highest risk of severe outcome before safe and effective vaccines and treatments are available. However, in contrast to SARS-CoV, viral shedding in patients with COVID-19 starts a few days before symptom onset (pre-symptomatic) and some patients remain asymptomatic while they shed the virus which make case isolation measures less efficient (Ganyani et al., 2020). In less than a year (as of December 11, 2020), a first vaccine based on a new platform consisting of mRNA encoding the spike protein of the virus (developed by Pfizer/BioNTech) has been granted for Emergency Use designation by the US FDA. Several other vaccines based on inactivated whole virus, recombinant non-replicative viral vector, recombinant spike protein and virus-like particles are in late phases of clinical investigation (Batty et al., 2020). The antiviral remdesivir (a repurposed drug initially developed for the treatment of Ebola virus) received US FDA Emergency Use Authorization for the treatment of hospitalized patients with COVID-19 requiring supplemental oxygen. The anti-inflammatory drug dexamethasone was recommended for critically ill patients with COVID-19 by the WHO (Lamontagne et al., 2020).

## How to Prevent a Future Pandemic?

Plague and cholera pandemics first disseminated along trade and military routes. Thereafter, the geographical spread of pathogens followed the movement of human population through rail, ship and air travels. Nowadays, the globalization of travels and trade of animals and animal-based foods further increase the spread of infectious diseases and the speed with which they are disseminated around the world. Land use and urbanization to accommodate agriculture and living areas modify the habitats of pathogens, hosts and disease vectors and affect the transmission dynamics of infections to humans. The geographic distribution of disease vectors and hosts as well as the living habitats of microorganisms is also affected by climate changes and could potentially increase the spread of pathogens. Increased contacts between humans and animals through breeding, hunting, wet markets and trade of exotic pets also favor the risk of spillover of zoonotic pathogens. The spread of infectious diseases is thus expected to increase due to human activities and their effects on the environment. Epidemics and pandemics will also occur more frequently and will represent new challenges for public health.

In order to control the transmission of water-borne pathogens such as *V. cholera*, the WHO has launched a water, sanitation and hygiene (WASH) program in developing countries (Matilla et al., 2018). This program emphasizes the provision of safe and clean sources of water, effective sanitation infrastructure and ensures appropriate hygiene practices. The WASH program was reported to increase safe drinking water accesses, safe sanitation services and basic handwashing facilities with soap and water at home to 71%, 45% and 60% of the global population, respectively (UNICEF and World Health Organization [WHO], 2019).

Vector control is the primary tool to control vector-borne diseases such as malaria, Dengue virus, Chikungunya virus and Zika virus (Wilson A.L. et al., 2020). These methods can target either the immature stages (by the use of predator species and chemical or biological larvicides or by the modification of the habitat) or the adult vectors (by the use of nets, topical repellents, insecticides and spraying). Furthermore, novel vector control methods are under development such as the genetic manipulation of mosquitos (Hammond and Galizi, 2017), bacterial infection of vectors (e.g., Wolbachia) (Flores and O’Neill, 2018) and eave tubes with insecticide-laden electrostatic netting (Knols et al., 2016). However, the development of novel vector control tools is still needed.

The implementation of global surveillance programs for the rapid detection of pathogen spillover from animal to the human population is of prime importance. The One Health concept promotes optimal health for human, animal and environment (One Health Commission [OHC], 2020). The environmental effects resulting from land use, urbanization and climate changes may increase the risk of pathogens spillover from animals to humans and emphasize the importance of a One Health integrative approach for the surveillance of zoonosis (Okello et al., 2011; Rabozzi et al., 2012). Such integrative approaches are used to implement surveillance programs for the prevention and control of emerging and re-emerging infections in developing countries. These multidisciplinary efforts could have positive impacts in these countries as they are the most afflicted by the effects of zoonoses (Bidaisee and Macpherson, 2014).

Viral zoonoses constitute particularly a serious threat to public health as viruses were at the origin of the most recent pandemics. It is thus important to evaluate the risk of cross-species transmission of viruses to humans. The viral richness associated to an animal species could predict zoonotic potential of mammalian viruses (Olival et al., 2017). It was shown that the cross-species transmission of mammalian viruses increases with respect to the phylogenetic proximity between hosts and humans. Furthermore, viruses that infect a phylogenetically broader ranges of hosts are more likely to be zoonotic. Characterizing the diversity of viruses in key wildlife species will help to reduce the time between detection and response during an outbreak (Epstein and Anthony, 2017). Furthermore, the global virome project was launched to detect and identify viral threats to human health, characterize the host ranges of viruses, identify behaviors that favor spillover, establish a global surveillance network and identify transmission and pathogenicity markers for high-risk viruses (Carroll et al., 2018; Kwok et al., 2020). Viral metagenomic next-generation sequencing analysis of nasal/throat swabs from individuals at risk of zoonotic infections was also used to expand the detection of novel viruses and the characterization of the respiratory virome of humans exposed to animals (Thi Kha Tu et al., 2020).

## Concluding Remarks

The time of onset and the pathogen that will cause the next pandemic are unpredictable. Therefore, pandemic preparedness plans emphasize that non-pharmaceutical interventions should be implemented first to control human-to-human transmission of the pathogen. Ideally, these interventions should adequately control the spread of an infection while minimizing societal and economic disruption. Risks of resurgence can follow once these non-pharmaceutical interventions are lifted. Once available, rapid testing together with contact tracing (Teixeira and Doetsch, 2020) and isolation of infected individuals should be put in place for a more effective response. Furthermore, pharmaceutical interventions including rapid point-of-care diagnostic tests (Hussein et al., 2020), biomarkers for disease stratification (Maertzdorf et al., 2016), broad spectrum antimicrobials/antivirals obtained through *in silico* drug repurposing (Mangione et al., 2020) or by the use of drugs targeting host cells (Lee and Yen, 2012) as well as new platforms for accelerated vaccine development and production (Rauch et al., 2018) should be developed to improve the global response to the pandemic.

## Author Contributions

JP wrote the manuscript. GB revised the manuscript. Both authors contributed to the article and approved the submitted version.

## Conflict of Interest

The authors declare that the research was conducted in the absence of any commercial or financial relationships that could be construed as a potential conflict of interest.
