# Corrigendum: Pandemics throughout history

**DOI:** 10.3389/fmicb.2022.988058

**Published:** 2022-09-27

**Authors:** Jocelyne Piret, Guy Boivin

**Affiliations:** CHU de Québec – Laval University, Quebec City, QC, Canada

**Keywords:** infectious diseases, zoonotic pathogens, pandemic, public health measures, pharmaceutical interventions, water-borne pathogens

In the original article, there was an error. The genera of two common human coronaviruses (HCoV-OC43 and HCoV-NL63) were inverted by mistake in two sentences.

A correction has been made to **Coronaviruses** section, first paragraph, pages 8–9. This sentence previously stated:

“HCoV-229E and HCoV-OC43, which belong to the alpha-coronavirus genus, are the causative agents of common cold (Kahn and McIntosh, 2005). HCoV-NL63 and HCoV-HKU1, which are members of the beta-coronavirus genus, cause more severe, although rarely fatal, infections of the upper and lower respiratory tracts (Kahn and McIntosh, 2005).”

The corrected sentence appears below:

“HCoV-229E (alpha-coronavirus) and HCoV-OC43 (beta-coronavirus) are the causative agents of common cold (Kahn and McIntosh, 2005). HCoV-NL63 (alpha-coronavirus) and HCoV-HKU1 (beta-coronavirus) cause more severe, although rarely fatal, infections of the upper and lower respiratory tracts (Kahn and McIntosh, 2005).”

The authors apologize for this error and state that this does not change the scientific conclusions of the article in any way. The original article has been updated.

## Publisher's note

All claims expressed in this article are solely those of the authors and do not necessarily represent those of their affiliated organizations, or those of the publisher, the editors and the reviewers. Any product that may be evaluated in this article, or claim that may be made by its manufacturer, is not guaranteed or endorsed by the publisher.
